# Identification of Potential Biomarkers and Survival Analysis for Head and Neck Squamous Cell Carcinoma Using Bioinformatics Strategy: A Study Based on TCGA and GEO Datasets

**DOI:** 10.1155/2019/7376034

**Published:** 2019-08-07

**Authors:** Yujie Shen, Jinhui Liu, Liqing Zhang, Shikun Dong, Jiacheng Zhang, Yaqin Liu, Han Zhou, Weida Dong

**Affiliations:** ^1^Department of Otorhinolaryngology, The First Affiliated Hospital of Nanjing Medical University, Nanjing 210029, Jiangsu, China; ^2^Department of Gynecology, The First Affiliated Hospital of Nanjing Medical University, Nanjing 210029, Jiangsu, China

## Abstract

The mechanism and gene markers of head and neck squamous cell carcinoma (HNSCC), a common malignant tumor, have not yet been identified. The aim of this study was to identify the key genes and pathways associated with HNSCC and to further analyze its molecular mechanism and prognostic significance. In this study, the expression profile chip data GSE6631 from Gene Expression Omnibus (GEO) included paired HNSCC tumor and normal samples from 22 patients; the RNAseq tertiary dataset of HNSCC and corresponding clinical information from The Cancer Genome Atlas (TCGA) included biological information of 12 normal head and neck tissues and 111 HNSCC sample tissues. Differentially expressed genes (DEGs) were screened by R software, and the pathway enrichment analysis of DEGs was performed by DAVID, String, and Sytoscape software programs. Combining the GEO and the TCGA databases, we used bioinformatics technology to screen out 50 DEGs in HNSCC and enrich the biological functions and key pathways of HNSCC. Then we performed Gene Ontology (GO) enrichment analysis, the Kyoto Encyclopedia of Genes and Genomes (KEGG) signaling pathway analysis, protein-protein interaction (PPI) analysis, and survival analysis on these DEGs. Using CMap, we identified candidate small molecules that might reverse HNSCC gene expression. Finally, four most important small molecules that could provide more reliable biomarkers for early diagnosis and individualized control of HNSCC were identified.

## 1. Introduction

Head and neck cancers rank in the top six malignancies worldwide, with approximately 600,000 new cases each year [1, 2]. Head and neck malignancies include tumors that originate in the mouth, nasopharynx, oropharynx, hypopharynx, larynx, and neck [3]. From the perspective of pathological classification, head and neck squamous cell carcinoma (HNSCC) is the main type of malignant tumor in the head and neck. According to the condition of human papillomavirus (HPV), HNSCC can be divided into two different subtypes: environmental carcinogen tobacco and alcohol-related and HPV-negative HNSCC [4], and HPV-positive HNSCC [5]. Head and neck tumors are clinically staged based on the size and location of the primary tumor (T), the number and size of cervical lymph node (N) metastases, and the presence or absence of distant metastases (M). The appropriate treatment plan (surgery, radiotherapy, chemotherapy) is selected according to the stage of the disease [6]. Though there are diverse treatments for HNSCC, patients have a low survival rate. To date, other than the HPV status, key biomarkers and precise targets for the development of HNSCC remain unclear.

In recent years, bioinformatics, a science combining molecular biology and information technology, has been used to study the molecular mechanisms of diseases by data mining at the molecular level [7] and discovered a large number of tumor markers that could be applied in clinical practice. These discoveries have showed great significance in revealing the molecular mechanism of tumor pathogenesis and remarkably improved the early diagnosis and prognosis of tumors.

Currently, microarray gene expression studies have been conducted to uncover molecular variations in a variety of tumors. Two data bases—Gene Expression Omnibus (GEO) and The Cancer Genome Atlas (TCGA)—serve to collect data from different microarray platforms. GEO, supported by the National Center for Biotechnology Information (NCBI) of the National Library of Medicine (NLM), accepts raw and processed data, including experimental designs for high-throughput gene expression and genomics research, written description of sample attributes, and methods [8]. The Cancer Genome Atlas (TCGA) project, supported by the National Institutes of Health, is a multi-agency innovation research project. TCGA currently collects and maintains genome-wide data, including encoded and noncoding RNA expression, somatic mutations, copy number changes, and promoter methylation, etc. [9].

**Figure 1:**
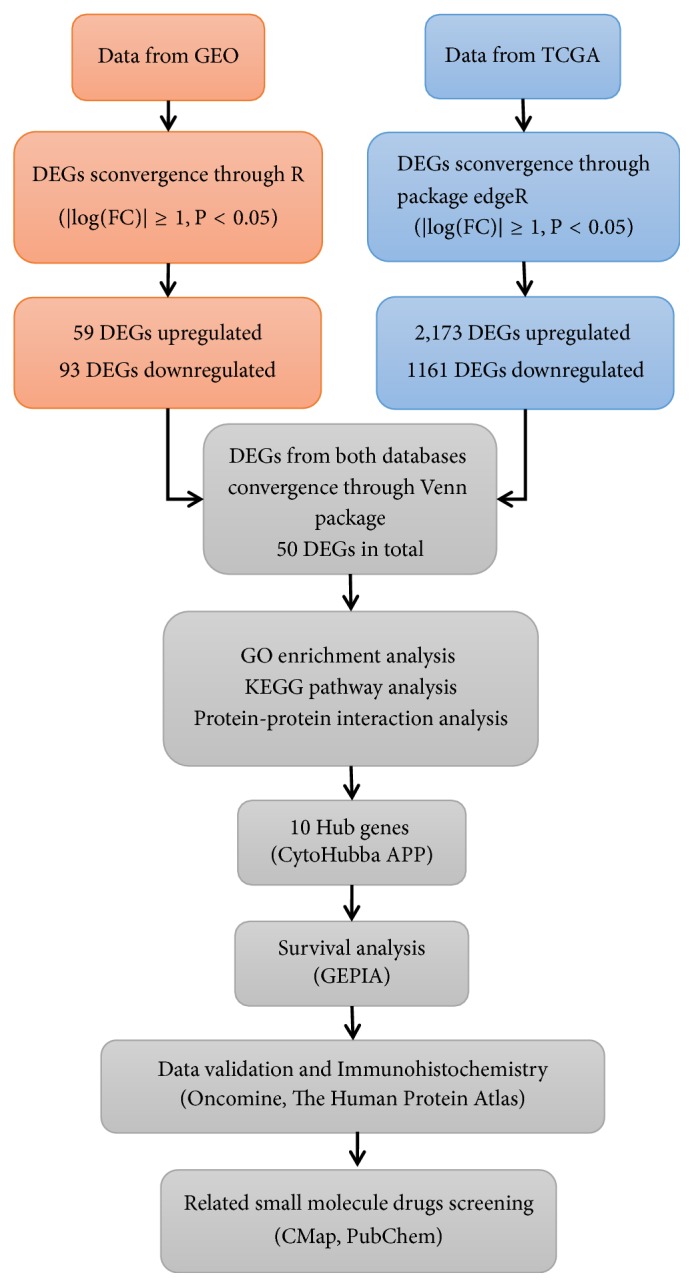
Flowchart for research into bioinformatics data from GEO and TCGA.

Tumor development is a complex pathological process involving multiple genetic alterations, including overexpression of oncogenes and/or inactivation of tumor suppressor genes [10]. In this study, bioinformatics methods and techniques were used to analyze and integrate the mRNA expression data of HNSCC from GEO and TCGA. Analyzing the molecular function of the integrated mRNA and the signaling pathways involved can further the understanding of the cause and potential molecular events of HNSCC. This study provides a reliable basis for exploring the molecular mechanisms of HNSCC pathogenesis and identifying molecular targets for clinical diagnosis or treatment. The workflow of the specific analysis is shown in Figure 1.

## 2. Materials and Methods

### 2.1. Microarray Datasets

In this study, we compared the gene expression profiles of the primary and adjacent normal tissues of HNSCC patients from GEO, downloaded and collected high-throughput gene expression dataset, numbered GSE6631[11]. Derived from the GPL8300 platform (Affymetrix Human Genome U95 Version 2 Array), this dataset includes paired HNSCC tumor and normal samples from 22 patients. The incision margin of the operation is about 1-3 cm. Normal tissue was taken from the contralateral mucosa or from an uninvolved site far from the tumor.17 sample pairs were obtained from patients undergoing surgery at New York University Medical Center, and 5 pairs from the Ninth People's Hospital in Shanghai, China. Among them, 13 samples were from oral cavity, 4 samples were from larynx,3 samples were from oropharynx,1 sample was from hypopharynx and 1 sample was from sinonasal cavity. At the same time, we downloaded 123 RNAseq dataset with mRNA level 3 and corresponding clinical information from 111 patients totally in TCGA, including biological information from 12 paired samples from tumor tissue and adjacent normal tissue, to calculate the expression of mRNA.

### 2.2. Data Processing and Differential Expressed Genes Analysis

Background correction, standardization and expression value calculation were performed on the original dataset GSE6631 from GEO using package Affy, Impute, and Limma of R software (version: x64 3.2.1). Fold-change (FC) and adjust p-values were used to screen differentially expressed genes (DEGs). |log⁡(FC)|≥ 1 and adj. p-val< 0.05 were defined as the screening criteria for DEGs. Here adj. p-val<0.05 was used in differential gene screening to control the false positive rate [12]. Heat and volcano maps of DEGs were constructed, using Pheatmap, Ggplot2 and other software packages. In the same way, we used the R software (version: x64 3.2.1) and the edgeR package to screen the DEGs in the TCGA dataset. The screening criteria were: |log⁡(FC)|≥ 1 and FDR<0.05. The heat and volcano maps were produced using the package gplots of R software. Subsequently, we took the intersected DEGs of the two datasets by the Venn package in the R software.

### 2.3. Function Enrichment Analysis of DEGs

GO provides a framework to describe the function of gene products in all organisms and identify characteristic biological properties or transcriptome data of high-throughput genomes [13]. GO annotation includes three categories: biological process (BP), cellular component (CC) and molecular function (MF). KEGG assigns specific paths of DEG sets to key data with higher-order functional information and can be used for functional interpretation and practical application of genomic information [14]. The Database for Annotation, Visualization, and Integrated Discovery (DAVID) 6.8 (https://david.ncifcrf.gov) is for gene function analysis. The analysis module provided by DAVID converts data to biological meaning and accelerates analysis of genome-level datasets [15]. To reveal the biological significance of the screened DEGs in HNSCC progression, we used DAVID to perform GO function and KEGG pathway enrichment analysis for the DEGs from the Venn package, and FDR < 0.05 was considered statistically significant.

### 2.4. PPI Analysis of DEGs

The interaction between proteins is essential to understanding the metabolic and molecular mechanisms of tumors. The String database (http://string-db.org) provides critical assessment and integration of protein interactions, including direct (physical) and indirect (functional) associations. The new version of the String10.0 database covers more than 2,000 organisms, almost twice of the previous version [16]. Protein-Protein Interaction (PPI) analysis of DEGs was performed using String10.0. The results of the analysis were imported into Cytoscape 3.7.1 to establish a network model. The plug-in app cytohubba in Cytoscape was downloaded and installed. The top ten scores of Maximal Clique Centrality (MCC) algorithm were taken as the criteria to screen out the Hub genes with high connectivity in the gene expression network.

### 2.5. Survival Analysis of Hub Genes

Gene Expression Profiling Interactive Analysis (GEPIA) (http://gepia.cancer-pku.cn) provides interactively analyses of cancer and normal genes [17]. To further clarify the relationship between Hub gene expression and HNSCC prognosis, we used GEPIA for survival analysis and log rank for statistical analysis. In log rank, p<0.05 was considered statistically significant. The Hub genes were taken as the key genes for HNSCC prognosis.

### 2.6. Verification of Key Genes

We used the online software Oncomine (https://www.oncomine.org) to verify the key genes [18]. In this study, we set the following screening criteria: 1 “Gene: key genes”; 2 “Analysis Type: Cancer vs. Normal Analysis”; 3 “Cancer Type: Head and Neck Cancer”; 4” Clinical Outcome: Survival Status “; 5 “Data Type: mRNA”. The key genes were verified by the following conditions: differential hub gene expression between head and neck tumor and normal tissues, the similar expression trend to our original databases, p< 0.05, Fold Change≥1.

### 2.7. Immunohistochemical Analysis

Based on the high specificity of binding between antibodies and antigens, immunohistochemistry can reveal the relative distribution and abundance of proteins. The Human Protein Atlas(HPA) (https://www.proteinatlas.org) is the largest and most comprehensive human tissue cell protein spatial distribution database[19], with enormous capabilities and potential application prospects. We used HPA to observe the differences in key gene expression between normal and HNSCC tissues.

### 2.8. Identification of Candidate Small Molecules

Connectivity Map (CMap) database (http://www.broadinstitute.org) predicts potential drugs in silicon that may reverse or induce biological states encoded in specific gene expression markers [20]. We compared the DEGs co-expressed by GEO and TCGA with data from CMap to predict small molecule activity that might reverse the biological state of HNSCC.

Firstly, the differential genes co-expressed obtained from the Venn package were divided into an up-regulated group and a down-regulated group, and then the two groups of genes were introduced into CMap for gene set enrichment analysis. Finally, small molecules with enrichment values ranging from - 1 to + 1 were obtained by calculation. Positive connectivity value (close to + 1) indicated that the corresponding small molecules could induce HNSCC gene expression, while negative connectivity value (close to - 1) indicated the greater similarity between genes and small molecules, which could reverse the state of HNSCC cells. The results were ranked by P value. We ranked the top four small molecules and analyzed their 3D Conformer in PubChem(http://www.pubchem.ncbi.nlm.gov), a public repository of small molecules and their biological properties [21].

## 3. Results

### 3.1. Identification of DEGs

**Figure 2:**
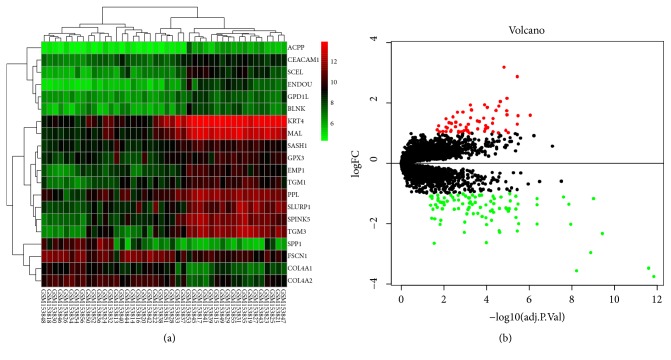
Heatmap and volcanic maps of 152 DEGs (including 59 up-regulated genes and 93 down-regulated genes) from GSE6631. (a) Singular samples: normal tissues; double samples: cancer tissue. Red: up-regulation; green: downregulation. (b) Red and green plots: differentially expressed mRNAs as indicated in (a); black plots: normally expressed mRNAs.

**Figure 3:**
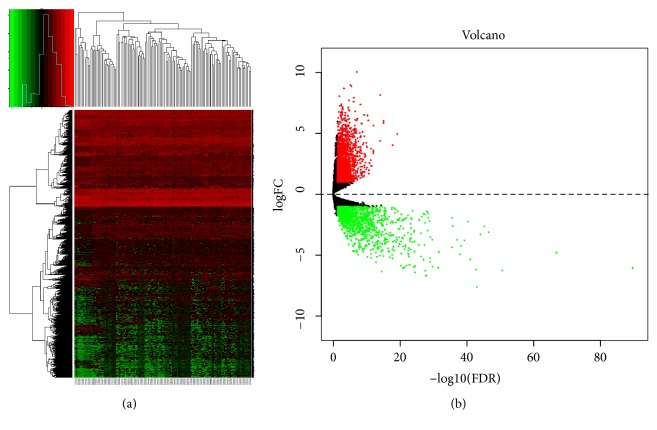
Heatmap and volcanic maps of 3334 DEGs (including 2173 up-regulated genes and 1161 down-regulated genes) from TCGA. (a) Red: upregulation; green: downregulation. (b) Red and green plots: differentially expressed mRNAs as indicated in (a); black plots: normally expressed mRNAs.

**Figure 4:**
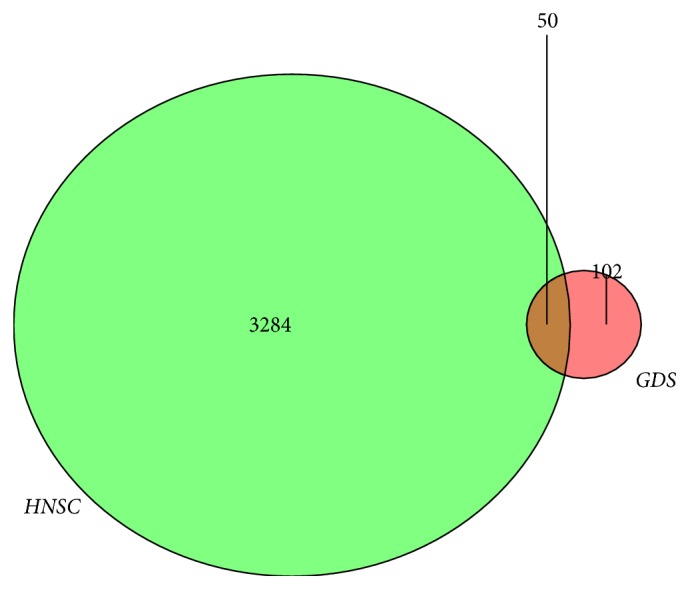
Venn diagram shows the intersecting DEGs from GEO and TCGA. Red area: GEO dataset; green area: TCGA dataset; cross area: DEGs expressed in both databases.

The expression profile chip data GSE6631 downloaded from GEO included paired HNSCC tumor and normal samples from 22 patients. According to the R software analysis and the screening conditions, 152 DEGs were extracted from the dataset GSE6631. Cluster analysis of these DEGs (Figure 2) showed 59 genes were upregulated and 93 genes were downregulated. The RNAseq tertiary data set of HNSCC from TCGA included the biological information of 12 normal head and neck tissues and 111 HNSCC samples. A total of 3334 DEGs were obtained from the TCGA database. Cluster analysis (Figure 3) revealed 2173 upregulated genes and 1161 downregulated genes. The Venn package was used to screen the intersecting DEGs from both databases, and generate the Venn map (Figure 4). Finally, 50 HNSCC-related DEGs with high reliability were obtained.

### 3.2. GO Enrichment Analysis of DEGs

**Figure 5:**
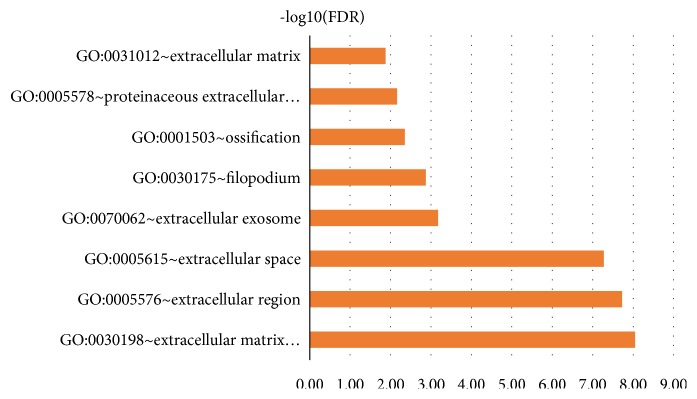
Significantly enriched GO terms of DEGs in HNSCC.

The GO enrichment analysis showed that the GO annotations of DEGs were divided into three parts: biological process (BP), cell composition (CC) and molecular function (MF). Terms were arranged in ascending order according to the FDR values. After screening, it was found that (FDR<0.05), and the DEGs were enriched in biological processes, such as extracellular matrix organization, extracellular region, extracellular space, extracellular exosome, filopodium, ossification, proteinaceous extracellular matrix, extracellular matrix (Table 1). The size of the -log10 (FDR) value was sorted, and the difference between the -log10 (FDR) values corresponding to the respective enrichment paths is shown in Figure 5.

### 3.3. KEGG Signaling Pathway Analysis of DEGs

**Figure 6:**
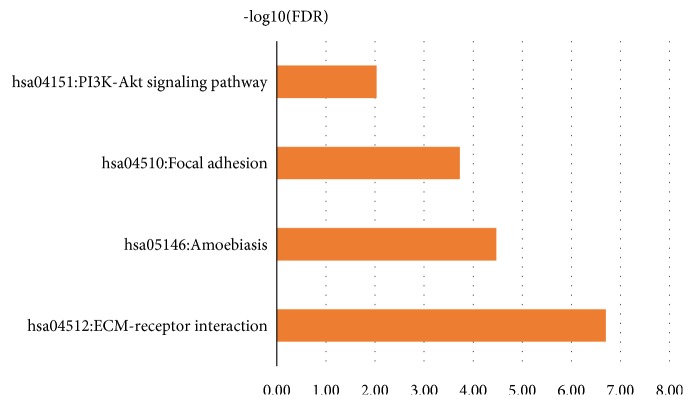
Significant KEGG pathway terms of DEGs in HNSCC.

The signal pathway results were ranked in ascending order according to the FDR value, and further screening was performed when FDR<0.05. The KEGG analysis showed that the DEGs were mainly concentrated in ECM-receptor interaction, Amoebiasis, Focal adhesion, PI3K-Akt signaling pathway and other signaling pathways (Table 2). According to the size of -log10 (FDR), the difference between the -log10 (FDR) values of the relevant signal paths is shown in Figure 6.

### 3.4. PPI Analysis of DEGs

**Figure 7:**
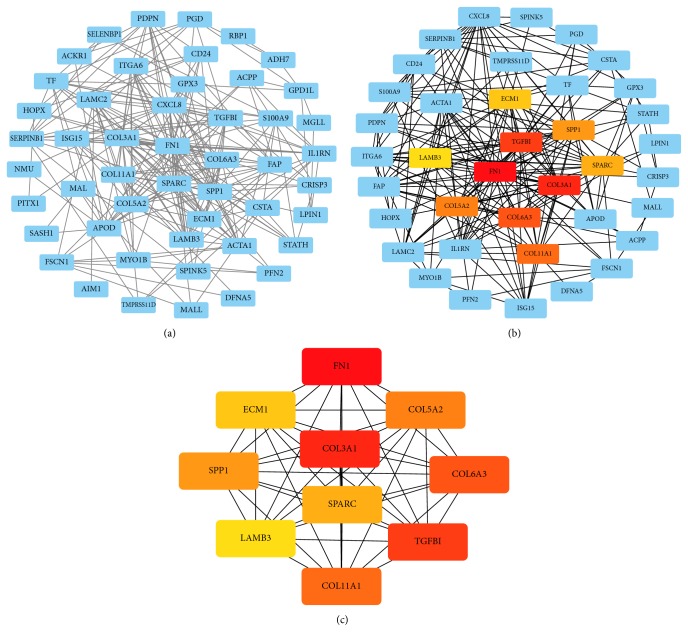
PPI analysis of DEGs based on Cytoscape. (a) Visualized PPI analysis of DEGs. (b) Top 10 genes with the highest MCC scores in DEGs. (c) Interconnection of 10 hub genes, darker color represents higher MCC scores.

The DEGs obtained were introduced into the online database String and the isolated genes without interaction were removed. Relevant PPI were obtained and visualized, revealing 50 nodes and 273 edges. The interacting genes were introduced into Cytoscape for network visualization analysis (Figure 7). Cytoscape and the plug-in app “cytoHubba” were used to parse the network. According to the “MCC” algorithm, the ten most stable and highest-scored genes (FN1, COL3A1, TGFBI, COL6A3, COL11A1, COL5A2, SPP1, SPARC, ECM1, and LAMB3) in the network were selected as the hub genes. (Figure 7).

### 3.5. Survival Analysis of Hub Genes

**Figure 8:**
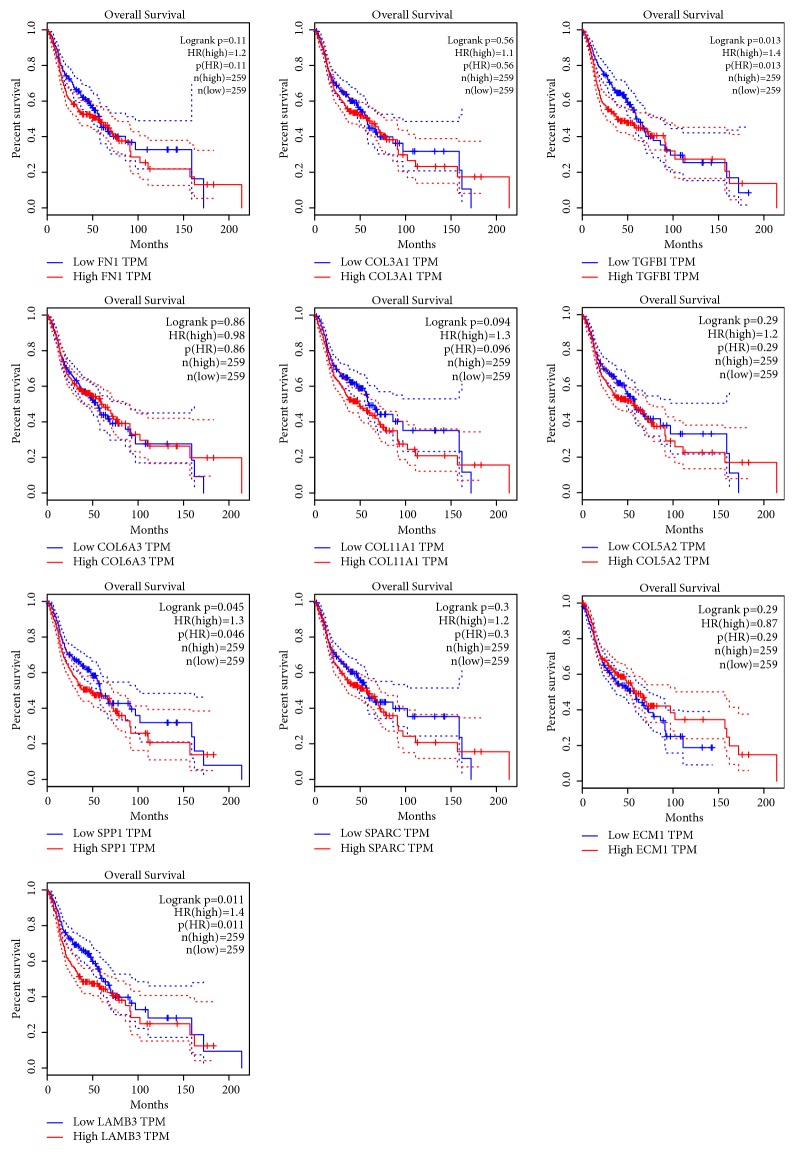
Survival analysis for hub genes in HNSCC. Survival curves show 10 genes related to overall survival rate (P<0.05).

When using GEPIA to analyze the correlation between hub gene expression and the prognosis of HNSCC, we found that the overall survival rate of high-expressing TGFBI was lower than that of low-expressing TGFBI (p=0.013); compared with low-expressing SPP1, the overall survival rate of high-expressing SPP1 was lower (p=0.045); compared with low-expressing LAMB3, the overall survival rate of high-expression group of LAMB3 was lower (p=0.011). No significant correlation was found between the expression of the other seven hub genes and HNSCC prognosis (p > 0.05), as shown in Figure 8. Therefore, TGFBI, SPP1, and LAMB3 were identified as three key genes.

### 3.6. Verification of Key Genes in the Oncomine Database

**Figure 9:**
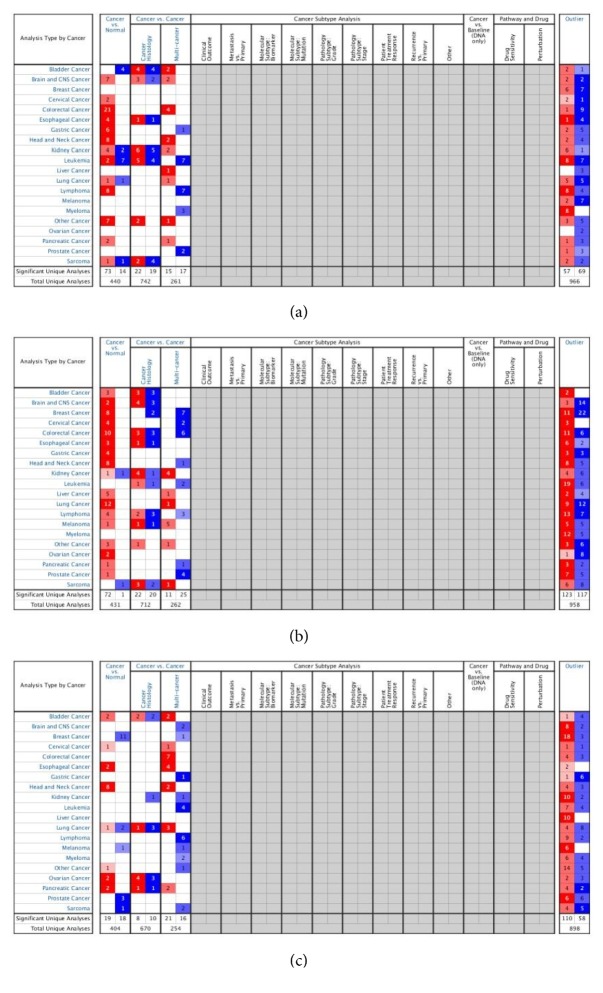
The mRNA expression of three key genes (Oncomine). (a) TGFBI mRNA expression was significantly higher in HNSCC than normal samples (b) SPP1 mRNA expression was significantly higher in HNSCC than normal samples. (c) LAMB3 mRNA expression was significantly higher in HNSCC than normal samples.

**Figure 10:**
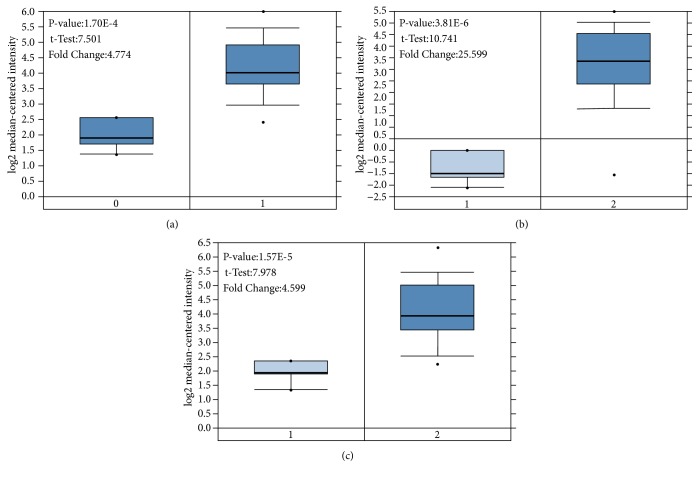
Analysis of TGFBI, SPP1, LAMB3 in HNSCC (Oncomine). Box plots: expression of the three Hub genes in normal and HNSCC tissues (p<0.01). (a) TGFBI mRNA expression (0: No value; 1: HNSCC). (b) TGFBI mRNA expression (1: Normal uvula; 2: HNSCC). (c) LAMB3 mRNA expression (1: Normal uvula; 2: HNSCC).

We validated the reliability of the key genes using Oncomine. The databases showed that the three key genes were differentially expressed in HNSCC and normal tissues (Figure 9), and all of them were highly expressed (p <0.01 and Ford Change> 4), as shown in Figure 10. By reviewing the original data, we found that three key genes were differentially expressed in Oncomine, GEO, and TCGA, and all of them showed high expression.

### 3.7. Immunohistochemical Analysis

**Figure 11:**
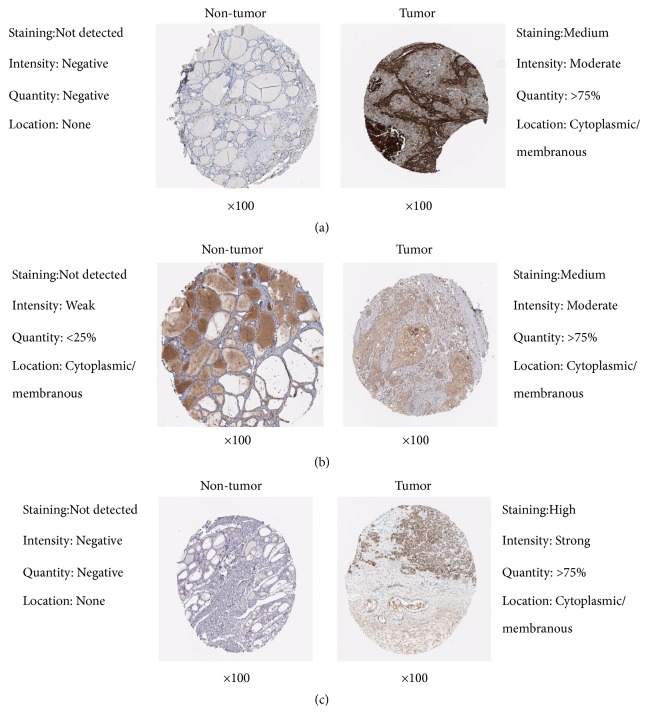
Elevated protein expression of TGFBI, SPP1, and LAMB3 in HNSCC samples from HPA. (a) TGFBI expression in normal neck tissues and HNSCC tissues from HPA. (b) SPP1 expression in normal neck tissues and HNSCC tissues from HPA. (c) LAMB3 expression in normal neck tissues and HNSCC tissues from HPA.

Since the protein data of the normal head and neck tissues are not listed separately in the Immunohistochemistry (IHC) database of HPA, we used the normal tissues from thyroid, a common site of head and neck tumors, as the control group. IHC database showed the protein expression of TGFBI, SPP1, and LAMB3 in head and neck tumor tissues were up-regulated compared with normal tissues (Figure 11). These results confirmed our findings.

### 3.8. Related Small Molecule Drugs Screening

**Figure 12:**
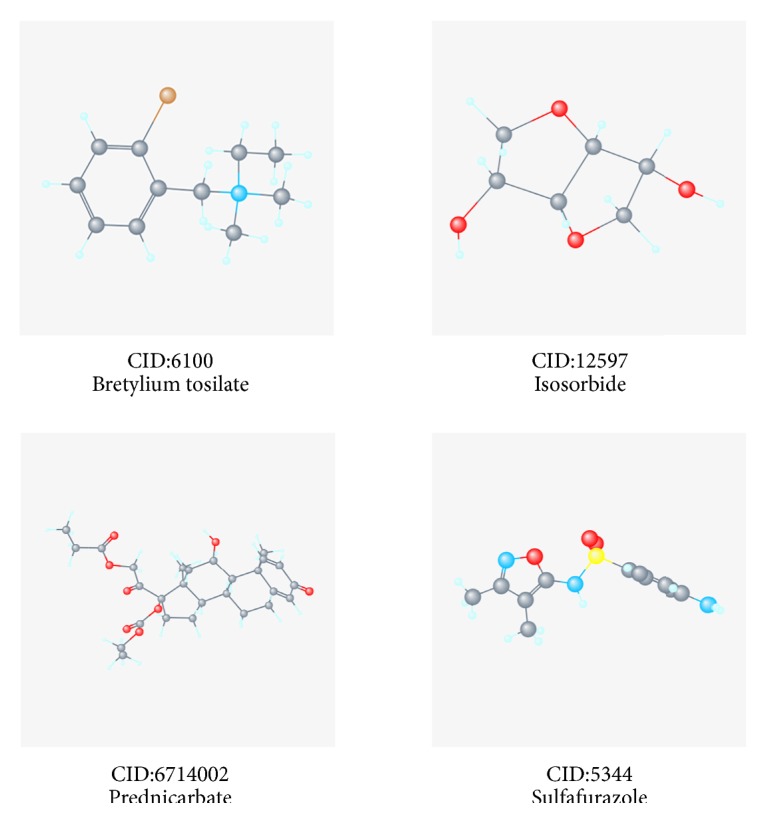
3D conformer of four most significant small molecule drugs.

To identify the potential drugs for HNSCC, we uploaded the up-regulated and down-regulated DEGs to the CMap database and matched them with small molecule therapy.  Table 3 lists the four most significant small molecule drugs and their enrichment values. Their 3D conformer (Figure 12) showed small molecules of bretylium tosilate (enrichment value -0.855) and prednicarbate (enrichment value -0.892) were associated with a highly significant negative fraction. These potential small molecule drugs could reverse the gene expression induced by HNSCC, providing guidance for developing targeted drugs for HNSCC. Further studies are still needed to confirm the value of these candidate small molecules in HNSCC treatment.

## 4. Discussion

The limitation of most previous studies is that they focused on a single genetic event or the results from a single cohort study. This study used bioinformatics to screen HNSCC-related genes based on the data of HNSCC gene expression in the GEO and TCGA databases. A total of 50 DEGs were analyzed by GO enrichment analysis and KEGG pathway. These genes functions in biological processes such as extracellular matrix organization, extracellular region, extracellular space, extracellular exosome, filopodium, ossification, proteinaceous extracellular matrix, and extracellular matrix. They are involved in ECM-receptor interaction, Amoebiasis, Focal adhesion, PI3K-Akt signaling pathway. The PPI network of these DEGs was analyzed by String10.0. After screening ten hub genes, three key genes (TGFBI, SPP1, LAMB3) related to HNSCC prognosis were identified. The Oncomine database was then used to verify the expression differences of these key genes between tumor and normal tissues. The combination of three databases (GEO, TCGA, and Oncomine) made our research results more credible, which was also a prominent feature of this study. Finally, immunohistochemical analysis and screening of potential small analytical drugs for HNSCC were carried out.

Transforming growth factor beta-induced protein (TGFBI), also known as betaig-3, is a multi-purpose matrix molecule induced by TGF-*β* [22]. This protein contains 4 fasciclin-1 domains that inhibit tumor growth and promote apoptosis [23, 24]. Most reports describe the role of TGFBI in corneal dystrophy [25] and in promoting glioma cell proliferation and migration [26]. In the present study, TGFBI was highly expressed in tumor tissues compared with adjacent non-tumor or normal tissues, showing a highest correlation with HNSCC. Studies have found that there are differences in the expression of TGFB1, SPP1 and other genes in oral squamous cell carcinoma (OSCC) [27]. This may be related to the involvement of TGFB1 in Adipose stromal cells-(ASCs-) mediated tumor fibrosis, extracellular matrix remodeling, and epithelial-mesenchymal transition (EMT)[28]. So far, there have been no reports on TGFB1 expression in other prone regions of head and neck tumors. Further validation of the role and association of TGFB1 and HNSCC development may lead to new targets in HNSCC treatment.

As the second most relevant gene in our study, secreted phosphoprotein 1 (SPP1), also known as Osteopontin (OPN), is located at 4q22.1. Participating in tumorigenesis and metastasis, SPP1 is overexpressed in a variety of malignancies, including lung adenocarcinoma [29] and gastric cancer [30]. OSCC is a subtype of HNSCC. Huang et al. [31] detected higher expression of SPP1, PAI and caveolin-1 in OSCC tissues than normal tissues by immunohistochemical staining, indicating that SPP1 overexpression is associated with OSCC carcinogenesis and progression, which is consistent with our findings. The expression of SPP1 in other subtypes of HNSCC has rarely been reported. Through the study of related literature, we found that miR-186 inhibits osteoarthritis chondrocyte apoptosis by interacting with SPP1 and regulating the Phosphatidylinositol‐ 3 kinase (PI3K)/protein kinase B (Akt) pathway. The present study also demonstrates that SPP1 is an important regulator of the PI3K-Akt pathway [32]. As an important signaling pathway related to HNSCC occurrence and development, PI3K-Akt highly affects cell progression, proliferation, migration and survival, and may be activated by G1/S of cell cycle [33], which is also closely related to the occurrence and development of tumors[34].

LAMB3 is one of the three subunits of laminin 332, a protein component of the extracellular matrix. In squamous cell carcinoma, laminin 332 promotes tumorigenesis through interaction with cell surface receptors [35]. At the same time, LAMB3 is an oncogene that participates in cancer cell migration and invasion. MIR-218 directly regulates lamb3 in HNSCC cells and promotes tumor cell migration and invasion via local adhesion pathways [36]. Liu et al. [37] demonstrates that LAMB3 is up-regulated in HNSCC cell lines and tissues. LAMB3 increases HNSCC cell migration and invasion by promoting epithelial-mesenchymal transition (EMT)-associated protein expression, rather than promoting proliferation. In addition, inhibition of LAMB3 increases cisplatin cytotoxicity of HNSCC cells. Studies have shown that LAMB3 leads to tumor invasion via Akt activation induced by the HGF/c-MET axis in papillary thyroid cancer [38]. In addition, there are also studies on the correlation between LAMB and the PI3K-Akt pathway [39].

HNSCC comprises a heterogeneous group of tumors that arise from the squamous epithelium of the mouth, nasopharynx, oropharynx, hypopharynx, larynx, and neck. Of course, differentially expressed genes may differ from subclinical subtypes. After the discussion in the last few paragraphs, in OSCC, the expression of TGFB1 and SPP1 were different and the expression of other subclinical sites of HNSCC was not reported.LAMB3 was upregulated in HNSCC patients but there is no specific head and neck subsites in the report. All of this suggests that we need to integrate more precise gene expression in subclinical sites of HNSCC on the basis of existing data because gene expression in different subclinical sites may also be different. Current studies have found that HPV is also closely related to HNSCC [40]. Reviewing the literature, we found that none of the three key genes was reported in HPV-positive HNSCC, further validation of the role and association of the three key genes and HPV-positive HNSCC development may lead to new targets in HPV-positive HNSCC treatment. Also, it may be considered that there is no significant correlation between the expression of these three key genes and the infection of HPV in HNSCC.

Consistent with our study, LI et al. [41] used bioinformatics methods to analyze mRNA of 43 normal samples and 498 HNSCC samples in the TCGA database. Among the 2594 identified DEGs, 1087 were up-regulated and 1507 were down-regulated. These DEGs were significantly enriched in the GOs and KEGG pathways of mitosis, cell cycle, Wnt, JAK/STAT and TLR signaling pathways. However, LI's research was only based on the TCGA dataset and has not been validated by other relevant databases and experiments. In the present study, we analyzed the datasets from both GEO and TCGA and took the intersection of the two databases to obtain more reliable DEGs. We used the online software GEPIA to perform survival analysis on DEGs, Oncomine online validation of key genes and immunohistochemical analysis of key genes, and used CMap to identify candidate small molecules that might reverse HNSCC gene expression.

In addition, we used the CMap database to identify a set of potential small molecules that mimic the expression of normal cellular genes. Small molecules with higher enrichment scores are more likely to reverse the HNSCC gene expression. This work may help to develop new targeted drugs for HNSCC. Bretylium tosilate (enriched fraction -0.855), a drug to combat arrhythmia, has not been reported as a therapy for HNSCC so far. Also, we found the small molecule prednicarbate (enriched fraction -0.892), a non-halogenated diester derivative of prednisone for inflammation and itching symptoms of corticosteroid-responsive dermatitis (such as atopic dermatitis)[42], has not been studied as a drug for HNSCC. Traditionally, treatment for head and neck squamous cell carcinoma (HNSCC) has included surgery, radiation, and chemotherapy depending on both the site and stage of disease. Although the continuous improvement of treatment methods, the management of recurrent and advanced disease is evolving. Therefore, further research is urgently needed to reveal the potential of chemomolecular targets in HNSCC treatment.

Reviewing the recent literature, we find that the overexpression of The epidermal growth factor receptor (EGFR) has been noted in up to 90% of HNSCC [43]and the EGFR mutation was associated with the grading and staging of HNSCC[44].EGFR inhibitors -monoclonal antibodies (mAbs) include cetuximab, panitumumab, zalutumumab and nimotuzumab. Among them, only cetuximab has been confirmed in the treatment of metastatic or recurrent HNSCC and was approved in combination with radiation by the US FDA for the treatment of unresectable HNSCC.EGFR inhibitors-tyrosine kinase inhibitors (TKIs), which consist of gefitinib, erlotinib and lapatinib, are also being investigated in the treatment of advanced/recurrent HNSCC, but there is no clear consensus on their true efficacy[45]. Vascular endothelial growth factor (VEGF) is a growth factor released by cells and stimulates angiogenesis, a key process in carcinogenesis. At present, there are three main types of VEGF inhibitors:(1)VEGFR inhibitors -monoclonal antibodies, such as bevacizumab;(2),VEGFR inhibitors-tyrosine kinase inhibitors, such as sunitinib; (3) other drugs that inhibit VEGFR, such as angiostatin and endostatin. What is more,it has been found that COX-2 participates in many processes of tumorigenesis and development, such as enhances invasiveness and metastasis of tumors, inhibits immunity and other physiological activities [46]. Activation of PI3K-AKT-mTOR pathway can enhance invasion and metastasis ability and reduce apoptosis of LSCC [47].In addition, Nuclear Factor Kappa B (NF-kB), Carbonyl reductase 1 (CBR1) and CD317 are also popular therapeutic targets for head and neck squamous cell carcinoma [48–50].

## 5. Conclusions

In summary, by comprehensively analyzing gene expression profiles, sequencing data of HNSCC and adjacent tissues and identifying key genes and important pathways in HNSCC occurrence and development, this study explains the pathogenesis and diagnosis of HNSCC from the perspective of bioinformatics, and also paves a new way for developing HNSCC targeted inhibitors. However, what remains unclear is the role of HNSCC-related signaling pathways, which needs further validation in clinical samples through molecular biology research and experiments such as RT-PCR and Western Blot.

## Figures and Tables

**Table 1:** GO analysis of DEGs (FDR<0.05).

Term	Description	Count	PValue	FDR	-log10(FDR)
GO:0030198	Extracellular matrix organization	12	6.41E-12	9.01E-09	8.05
GO:0005576	Extracellular region	23	1.64E-11	1.88E-08	7.73
GO:0005615	Extracellular space	21	4.65E-11	5.35E-08	7.27
GO:0070062	Extracellular exosome	23	5.83E-07	6.71E-04	3.17
GO:0030175	Filopodium	6	1.17E-06	0.001344444	2.87
GO:0001503	Ossification	6	3.15E-06	0.004434985	2.35
GO:0005578	Proteinaceous extracellular matrix	8	6.05E-06	0.006955342	2.16
GO:0031012	Extracellular matrix	8	1.16E-05	0.01329234	1.88

**Table 2:** KEGG pathway analysis of DEGs in HNSCC(FDR<0.05).

Category	Term	Count	PValue	FDR	-log10(FDR)
KEGG_PATH WAY	hsa04512:ECM-receptor interaction	9	1.98E-10	1.99E-07	6.70
KEGG_PATH WAY	hsa05146:Amoebiasis	8	3.38E-08	3.39E-05	4.47
KEGG_PATH WAY	hsa04510:Focal adhesion	9	1.88E-07	1.89E-04	3.72
KEGG_PATH WAY	hsa04151:PI3K-Akt signaling pathway	9	9.33E-06	0.009344962	2.03

**Table 3:** Four most significant small molecule drugs.

Rank	CMap name	Mean	N	Enrichment	P	CID
1	bretylium tosilate	-0.526	4	-0.855	0.0008	6100
2	isosorbide	0.588	4	0.846	0.0008	12597
3	prednicarbate	-0.674	3	-0.892	0.0024	6714002
4	sulfafurazole	0.366	5	0.735	0.003	5344

## Data Availability

The data used to support the findings of this study are available from the corresponding author upon request.

## References

[1] Shield K. D. (2012). The global incidence of lip, oral cavity, and pharyngeal cancers by subsite in. *A Cancer Journal for Clinicians*.

[2] Torre L. A., Bray F., Siegel R. L., Ferlay J., Lortet-Tieulent J. (2015). Global cancer statistics, 2012. *CA: A Cancer Journal for Clinicians*.

[3] Marur S., Forastiere A. A. (2016). Head and Neck Squamous Cell Carcinoma: Update on Epidemiology, Diagnosis, and Treatment. *Mayo Clinic Proceedings*.

[4] Jethwa A. R., Khariwala S. S. (2017). Tobacco-related carcinogenesis in head and neck cancer. *Cancer and Metastasis Reviews*.

[5] Vokes E. E., Agrawal N., Seiwert T. Y. (2015). HPV-Associated Head and Neck Cancer. *Journal of the National Cancer Institute*.

[6] Specenier P., Vermorken J. B. (2018). Optimizing treatments for recurrent or metastatic head and neck squamous cell carcinoma. *Expert Review of Anticancer Therapy*.

[7] Li S., Yang R., Sun X. (2018). Identification of SPP1 as a promising biomarker to predict clinical outcome of lung adenocarcinoma individuals. *Gene*.

[8] Clough E., Barrett T. (2016). The gene expression omnibus database. *Methods in Molecular Biology*.

[9] Lee J.-S. (2016). Exploring cancer genomic data from the cancer genome atlas project. *BMB Reports*.

[10] Vogelstein B., Kinzler K. W. (2004). Cancer genes and the pathways they control. *Nature Medicine*.

[11] Kuriakose M. A. (2004). Selection and validation of differentially expressed genes in head and neck cancer. *Cellular and Molecular Life Sciences*.

[12] Noble W. S. (2009). How does multiple testing correction work?. *Nature Biotechnology*.

[13] Thomas P. D. (2017). The Gene Ontology and the Meaning of Biological Function. *Methods in Molecular Biology*.

[14] Kanehisa M., Furumichi M., Tanabe M., Sato Y., Morishima K. (2017). KEGG: new perspectives on genomes, pathways, diseases and drugs. *Nucleic Acids Research*.

[15] Dennis G. J. (2003). DAVID: Database for Annotation, Visualization, and Integrated Discovery. *Genome Biology*.

[16] Szklarczyk D., Franceschini A., Wyder S. (2015). STRING v10: protein-protein interaction networks, integrated over the tree of life. *Nucleic Acids Research*.

[17] Tang Z., Li C., Kang B., Gao G., Li C., Zhang Z. (2017). GEPIA: A web server for cancer and normal gene expression profiling and interactive analyses. *Nucleic Acids Research*.

[18] Rhodes D. R., Yu J., Shanker K. (2004). ONCOMINE: a cancer microarray database and integrated data-mining platform. *Neoplasia*.

[19] Thul P. J., Lindskog C. (2018). The human protein atlas: A spatial map of the human proteome. *Protein Science*.

[20] Lamb J., Crawford E. D., Peck D. (2006). The connectivity map: using gene-expression signatures to connect small molecules, genes, and disease. *Science*.

[21] Li Q., Cheng T., Wang Y., Bryant S. H. (2010). PubChem as a public resource for drug discovery. *Drug Discovery Therapy*.

[22] Thapa N., Lee B.-H., Kim I.-S. (2007). TGFBIp/*β*ig-h3 protein: a versatile matrix molecule induced by TGF-*β*. *The International Journal of Biochemistry & Cell Biology*.

[23] Skonier J., Neubauer M., Madisen L., Bennett K., Plowman G. D., Purchio A. (1992). cDNA Cloning and Sequence Analysis of *β*ig-h3, a Novel Gene Induced in a Human Adenocarcinoma Cell Line after Treatment with Transforming Growth Factor-*β*. *DNA and Cell Biology*.

[24] Zhao Y. L., Piao C. Q., Hei T. K. (2002). Overexpression of Betaig-h3 gene downregulates integrin *α*5*β*1 and suppresses tumorigenicity in radiation-induced tumorigenic human bronchial epithelial cells. *British Journal of Cancer*.

[25] Han K. E., Choi S., Kim T. (2016). Pathogenesis and treatments of TGFBI corneal dystrophies. *Progress in Retinal and Eye Research*.

[26] Guo S. K. (2018). Enhanced Expression of TGFBI Promotes the Proliferation and Migration of Glioma Cells. *Cellular Physiology and Biochemistry*.

[27] Hu Q., Peng J., Chen X. (2019). Obesity and genes related to lipid metabolism predict poor survival in oral squamous cell carcinoma. *Oral Oncology*.

[28] Donohoe C. L., Lysaght J., O'Sullivan J., Reynolds J. V. (2017). Emerging Concepts Linking Obesity with the Hallmarks of Cancer. *Trends in Endocrinology & Metabolism*.

[29] Li Y., Gu J., Xu F., Zhu Q., Ge D., Lu C. (2018). Transcriptomic and functional network features of lung squamous cell carcinoma through integrative analysis of GEO and TCGA data. *Scientific Reports*.

[30] Zhuo C., Li X., Zhuang H. (2016). Elevated THBS2, COL1A2, and SPP1 Expression Levels as Predictors of Gastric Cancer Prognosis. *Cellular Physiology and Biochemistry*.

[31] Huang C. F. (2014). Prognostic and predictive values of SPP1, PAI and caveolin-1 in patients with oral squamous cell carcinoma. *International Journal of Clinical and Experimental Pathology*.

[32] Lin Z., Tian X., Huang X., He L., Xu F. (2019). microRNA-186 inhibition of PI3K-AKT pathway via SPP1 inhibits chondrocyte apoptosis in mice with osteoarthritis. *Journal of Cellular Physiology*.

[33] Prasad S. B., Yadav S. S., Das M. (2015). PI3K/AKT pathway-mediated regulation of p27Kip1 is associated with cell cycle arrest and apoptosis in cervical cancer. *Cellular Oncology*.

[34] Fresno Vara J. A., Casado E., de Castro J., Cejas P., Belda-Iniesta C., González-Barón M. (2004). PI3K/Akt signalling pathway and cancer. *Cancer Treatment Reviews*.

[35] Ortiz-Urda S. (2005). Type VII collagen is required for Ras-driven human epidermal tumorigenesis. *Science*.

[36] Kinoshita T., Hanazawa T., Nohata N. (2012). Tumor suppressive microRNA-218 inhibits cancer cell migration and invasion through targeting laminin-332 in head and neck squamous cell carcinoma. *Oncotarget *.

[37] Liu L., Jung S., Oh C. (2019). LAMB3 is associated with disease progression and cisplatin cytotoxic sensitivity in head and neck squamous cell carcinoma. *European Journal of Surgical Oncology*.

[38] Jung S. N. (2018). LAMB3 mediates metastatic tumor behavior in papillary thyroid cancer by regulating c-MET/Akt signals. *Scientific Reports*.

[39] Zhang H., Pan Y., Cheung M. (2019). LAMB3 mediates apoptotic, proliferative, invasive, and metastatic behaviors in pancreatic cancer by regulating the PI3K/Akt signaling pathway. *Cell Death & Disease*.

[40] Aggarwal C. (2016). DNA-based immunotherapy for HPV-associated head and neck cancer. *Immunotherapy*.

[41] Yan L., Zhan C., Wu J., Wang S. (2016). Expression profile analysis of head and neck squamous cell carcinomas using data from the cancer genome atlas. *Molecular Medicine Reports*.

[42] Gupta A. K., Chow M. (2004). Prednicarbate (Dermatop): Profile of a corticosteroid. *Journal of Cutaneous Medicine and Surgery*.

[43] Kalyankrishna S., Grandis J. R. (2006). Epidermal Growth Factor Receptor Biology in Head and Neck Cancer. *Journal of Clinical Oncology*.

[44] Vatte C., Al Amri A. M., Cyrus C. (2017). Tyrosine kinase domain mutations of <em>EGFR</em> gene in head and neck squamous cell carcinoma. *OncoTargets and Therapy*.

[45] Azoury S. C., Gilmore R. C., Shukla V. (2016). Molecularly targeted agents and immunotherapy for the treatment of head and neck squamous cell cancer (HNSCC). *Discovery Medicine*.

[46] Bonhin R. G., de Carvalho G. M., Guimaraes A. C. (2017). Histologic correlation of VEGF and COX-2 expression with tumor size in squamous cell carcinoma of the larynx and hypopharynx. *Ear, Nose & Throat Journal*.

[47] Wang B., Qin H., Wang Y. (2014). Effect of DJ-1 overexpression on the proliferation, apoptosis, invasion and migration of laryngeal squamous cell carcinoma SNU-46 cells through PI3K/AKT/mTOR. *Oncology Reports*.

[48] Monisha J., Kishor Roy N., Bordoloi D. (2016). Nuclear Factor Kappa B: A Potential Target to Persecute Head and Neck Cancer. *Current Drug Targets*.

[49] Yun M., Choi A. J., Lee Y. C. (2018). Carbonyl reductase 1 is a new target to improve the effect of radiotherapy on head and neck squamous cell carcinoma. *Journal of Experimental & Clinical Cancer Research*.

[50] Yang L., Wu L., Yu G., Zhang W., Liu B., Sun Z. (2018). CD317 Signature in Head and Neck Cancer Indicates Poor Prognosis. *Journal of Dental Research*.

